# On leveraging self-supervised learning for accurate HCV genotyping

**DOI:** 10.1038/s41598-024-64209-y

**Published:** 2024-07-05

**Authors:** Ahmed M. Fahmy, Muhammed S. Hammad, Mai S. Mabrouk, Walid I. Al-atabany

**Affiliations:** 1 Computer Science program, School of Information Technology and Computer Science (ITCS), Nile University, Sheikh Zayed City, Egypt; 2 Biomedical Engineering Department, Faculty of Engineering, Helwan University, Cairo, Egypt; 3 Biomedical informatics program, School of Information Technology and Computer Science (ITCS), Nile University, Sheikh Zayed City, Egypt

**Keywords:** Image processing, Machine learning

## Abstract

Hepatitis C virus (HCV) is a major global health concern, affecting millions of individuals worldwide. While existing literature predominantly focuses on disease classification using clinical data, there exists a critical research gap concerning HCV genotyping based on genomic sequences. Accurate HCV genotyping is essential for patient management and treatment decisions. While the neural models excel at capturing complex patterns, they still face challenges, such as data scarcity, that exist a lot in computational genomics. To overcome this challenges, this paper introduces an advanced deep learning approach for HCV genotyping based on the graphical representation of nucleotide sequences that outperforms classical approaches. Notably, it is effective for both partial and complete HCV genomes and addresses challenges associated with imbalanced datasets. In this work, ten HCV genotypes: 1a, 1b, 2a, 2b, 2c, 3a, 3b, 4, 5, and 6 were used in the analysis. This study utilizes Chaos Game Representation for 2D mapping of genomic sequences, employing self-supervised learning using convolutional autoencoder for deep feature extraction, resulting in an outstanding performance for HCV genotyping compared to various machine learning and deep learning models. This baseline provides a benchmark against which the performance of the proposed approach and other models can be evaluated. The experimental results showcase a remarkable classification accuracy of over 99%, outperforming traditional deep learning models. This performance demonstrates the capability of the proposed model to accurately identify HCV genotypes in both partial and complete sequences and in dealing with data scarcity for certain genotypes. The results of the proposed model are compared to NCBI genotyping tool.

## Introduction

Hepatitis C virus (HCV) is a global pandemic affecting over 185 million people worldwide^1^. HCV is a member of the Flaviviridae family. Its genetic material consists of single-strained RNA of approximately 9.6 kb^2^. HCV leads to chronic disease and is a leading cause of liver transplantation, particularly impacting those coinfected with human immunodeficiency virus(HIV)^3^. HCV treatment is a top priority due to the potential for rapid progression of HCV in HIV-positive patients^4^. Coinfection accelerates liver disease progression^5,6^, leading to hospitalization and death^7^. HCV has various genotypes and subtypes; It is categorized into seven main genotypes and subdivided into 64 distinct subtypes based on genetic diversity^8^.

Furthermore, there is a wide variation in HCV genotype distribution in different regions worldwide. HCV genotype 1 is the most widespread worldwide, representing 46% of cases. In North America, Europe, and Australia, the prevalent HCV subtypes are 1a and 1b, whereas in Japan, subtype 1b infection is found in 73% of HCV-infected individuals. The second most common HCV genotype worldwide is genotype 3, with a prevalence of 30% and predominantly found in South Asia. Genotype 4 is primarily concentrated in Africa and the Middle East, while genotypes 5 and 6 are exclusive to Southern Africa and Southeast Asia, respectively^9,10^. It was found that the risk for Hepatocellular carcinoma (HCC) development is prevalent among patients infected with HCV type 1b compared to those with type 2a and 2c infections. Additional factors contributing to HCC included gender, excessive alcohol consumption, age over 60, and the absence of interferon treatment. Cirrhosis Patients infected with HCV type 1b have a notably higher risk of developing HCC in comparison to those with different HCV genotypes^11^.

HCV genotyping plays a critical role in guiding treatment decisions for chronic hepatitis C virus (HCV) infection. Identifying the specific HCV genotype in an individual is essential for selecting the most appropriate direct-acting antiviral (DAA) regimen^12,13^. Additionally, HCV genotyping can make a noticeable contribution as follows, Different HCV genotypes may respond differently to antiviral treatments. Therefore, Accurate genotyping and subtyping of HCV is very important, especially for deciding appropriate antiviral therapy^8^. Since Some genotypes are associated with a higher risk of liver disease progression and complications, including hepatocellular carcinoma (liver cancer), genotyping can provide insights into the patient’s prognosis.

From another perspective, understanding the geographic distribution of HCV genotypes is critical for public health surveillance and intervention strategies. Certain genotypes may be more common in specific regions or populations due to factors such as historical migration, healthcare practices, and social behaviors. Epidemiological studies of HCV genotypes provide insights into transmission dynamics, risk factors for infection, and the impact of geographic factors on viral diversity. Traditional HCV genotyping methods are constrained by certain limitations including: accurate genotype identification^8,14^, contamination risks, and technical challenges^15^. These considerations led to the primary focus of this study on predicting HCV genotype. We summarize our contributions as follows:

1. Proposing Advanced Deep Learning Approach for HCV genotyping based on convolutional auto-encoders.
2. Genotyping of both Partial and Complete HCV sequences.
3. Addressing Imbalanced genotypes distribution.

The remaining part of this paper is organized as follows: “Literature review” section introduces the literature review of HCV genotyping. Moving forward to “Research methodology” section, we discuss the methodologies used in this work comprising dataset collection, comparative analysis, sequences preprocessing and encoding, cross validation, classification and evaluation. After that, “Results and discussion” section discusses the results of our work. Finally, “Conclusion” section concludes the paper.

## Literature review

HCV genotypes exhibit approximately 31–33% nucleotide-level differences, while subtypes show variations of about 20–25%. Despite the genetic diversity of HCV, specific subgenomic regions, including HCV 5ʹNCR, core, E1, and NS5b, are employed by researchers for genotyping purposes^8^. Currently, various HCV genotyping tests are used in clinical laboratories for detecting the genotypes of the Hepatitis C virus, including PCR-RFLP, PCR using genotype-specific primers, etc. These methods have some limitations in accurately identifying some genotypes of HCV. For example, In a study conducted in South India aiming to compare different hepatitis C virus genotyping methods^8^, HCV genotyping was carried out using two methods: core region type-specific PCR and 5$^{\prime }$NCR PCR-RFLP. The results displayed good overall agreement between the various genotyping methods. However, the 5$^{\prime }$NCR PCR-RFLP method was able to correctly identify only 14% of HCV genotype 6 samples, incorrectly classifying the remaining 86% as genotype 1.

Likewise, core type-specific PCR method was able to correctly identify only 57.1% of HCV genotype 6 samples. The same problem of misclassification of HCV genotype 6 was reported in this study^14^, mentioning that the tested 5ʹNC methods should not be used for subtyping. Other challenges include Contamination with previously amplified material, which can lead to false positive results. Additionally, mistaken classifications can be occurred through cross-reactivity^16^. Furthermore, these tests can be time-consuming and technically challenging^15^. Another studies demonstrated that RT-PCR tests have high false postives and false negatives^17,18^. So, While these HCV genotyping tests offer valuable benefits and are currently used for diagnosing HCV in patients, these methods may require further refinement to reduce cross-reactivity and improve the detection of minor genotypes in clinical settings.

To address these concerns, multiple studies focusing on analyzing and classifying various viruses, have emphasized the imperative role of computational approaches. This shift is driven by the understanding that computational approaches not only sidestep many issues linked to wet-lab techniques but also provide a more systematic, scalable, and error-resistant approach to analysis. Adopting computational methods in virus research is a strategic move to enhance precision, minimize experimental biases, and boost the overall reliability of findings in the ever-evolving field of virology. Researchers across various studies increasingly turn to computational methods, particularly machine learning, to enhance the understanding and diagnosis of different viruses, showcasing a growing trend in the scientific community.

The current studies focus on HCV identification using clinical data. Clinical data refers to the information collected during the diagnosis and treatment of patients in a clinical setting. This data includes a wide range of information such as medical history, laboratory test results, imaging studies, and vital signs. By analyzing large sets of clinical data, researchers can identify patterns and trends that can help in the early detection and diagnosis of diseases as well as risk factors.

Several studies have explored the application of machine learning in predicting HCV, focusing on diverse datasets and methodologies. Aseem et al.^19^ compared different classifiers using datasets from the UCI ML repository and GitHub, finding that Random Forest (RF) achieved the highest accuracy of 71–72%. Jadhav^20^ developed a secure framework for HCV prediction, integrating Random Forest and AdaBoost to achieve 88.7% accuracy. Akter et al.^21^ classified Hepatitis C individuals with logistic regression (95% accuracy), SVM (95% accuracy), and XGBoost (92% accuracy). Edeh et al.^22^ explored an ensemble model for Hepatitis C prediction, achieving 95.59% accuracy. Safdari et al.^23^ used RF with SMOTE to reach 97.29% accuracy. Alizargar^24^ employed SVM and XGBoost with up to 95% accuracy. Lilhore^25^ proposed a Hybrid Predictive Model (HPM) with 96.82% accuracy, outperforming standard ML models. Li^26^ introduced Stepwise Random Forests and Logistic Regression (98.74% accuracy). While Fan et al.^27^ presented IHCP, achieving 99.44% accuracy with RF. These studies collectively demonstrate the efficacy of machine learning in medical diagnostics, emphasizing the importance of classifier selection in achieving accurate predictions for HCV. Notably, these studies primarily focus on predicting HCV based on clinical data, without addressing HCV genotyping objective.

To the best of our knowledge, only one study used the nucleotide sequences for HCV genotyping. Qiu et al.^28^ developed a fast and accurate method for determining the genotype of Hepatitis C Virus (HCV) strains, which is crucial for clinical management and patient response to antiviral therapy. The study utilized a global Position Weight Matrix (PWM) for the HCV genome to select genotype-specific nucleotide sequence “signatures” including, 5ʹ NCR, CORE, E1, and NS5B, from different regions of the HCV genome. Two discriminant methods, support vector machine (SVM) and random forest (RF), were used to build predictive models. The study used publicly available HCV nucleotide sequences from National Center for Biotechnology Information (ncbi). The results reveals that models built based on features from NS5B and E1 perform better than those based on features from CORE and 5ʹ NCR. The above study focused on HCV genotyping by selecting specific nucleotide positions based on conservation information and using them for model developement. However, this approach has limitations compared to our method. In our study, we eliminate the need to select specific regions of the HCV genome, This allows for a more comprehensive analysis and reduces the risk of overlooking important genetic variations that may occur in regions not specifically targeted for selection. Additionally, our dataset is substantially larger, comprising 51,092 samples, compared to the 10,014 samples used in the previous study. This larger dataset provides a more extensive and diverse set of genetic information, potentially leading to more robust and accurate genotyping results. The third limitation in the mentioned study is that it eliminated partial sequences missing more than one third of the selected positions from both the training and prediction sets. Contrary to our approach, as we are able to utilize partial sequences more effectively.

Multiple studies focused on applying genomic image processing (GIP) techniques for detecting and analyzing viruses. A study by Tanchotsrinon et al.^29^ introduces two new feature extraction techniques, ChaosCentroid and ChaosFrequency, for predicting HPV genotypes associated with cancer. The study examines 12 HPV genotypes and proposes the use of Chaos Game Representation (CGR) to represent HPV genomes. The study of Hammad et al.^30^ proposed a hybrid approach based on genomic image processing techniques to rapidly detect COVID-19 and other HCoV diseases. By converting genome sequences into genomic grayscale images and extracting deep features using a pre-trained convolutional neural network.

## Research methodology

**Figure 1:**
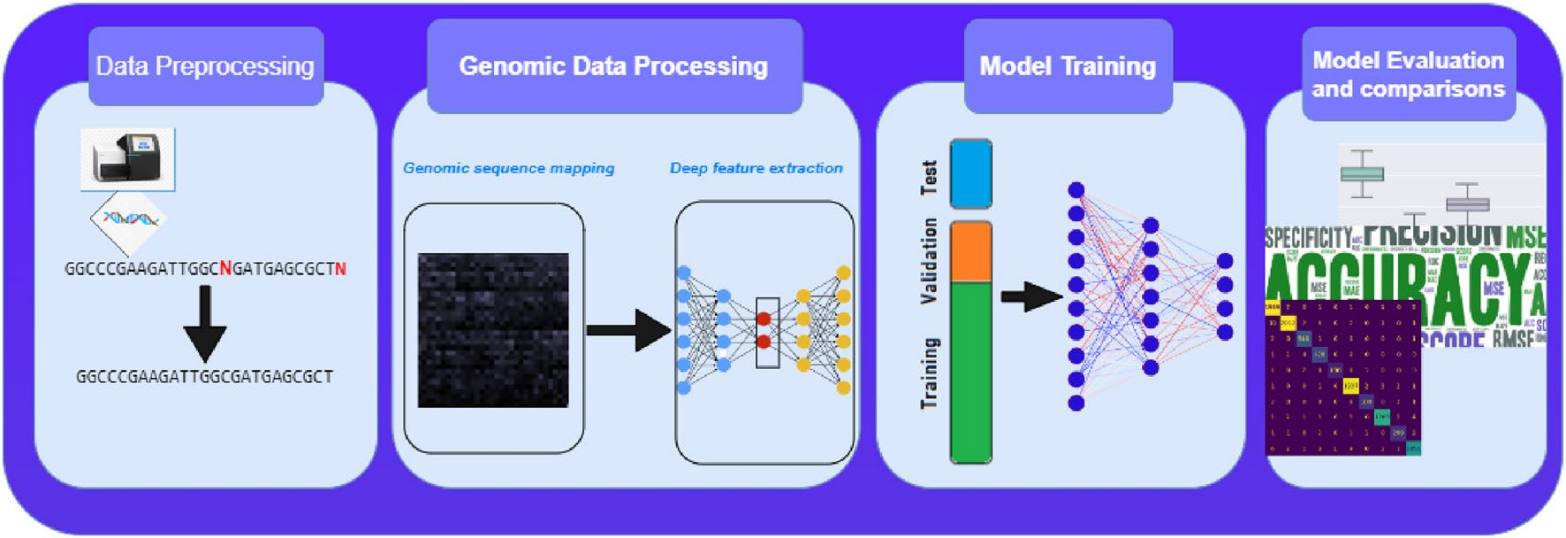
The proposed system pipeline.

Our proposed system of HCV genotyping involves a comprehensive seven-phase system. The methodology encompasses dataset preparation, genomic sequence preprocessing, transforming the sequences into coordinates in chaos game representation (CGR), and Feature extraction using autoencoder. After that, the extracted features were tested on Convolutional Neural Network. Lastly, the output from the test set was used to evaluate the system’s performance and compare it with different models. Figure 1 shows the pipeline of the proposed system.

### Dataset collection

**Figure 2:**
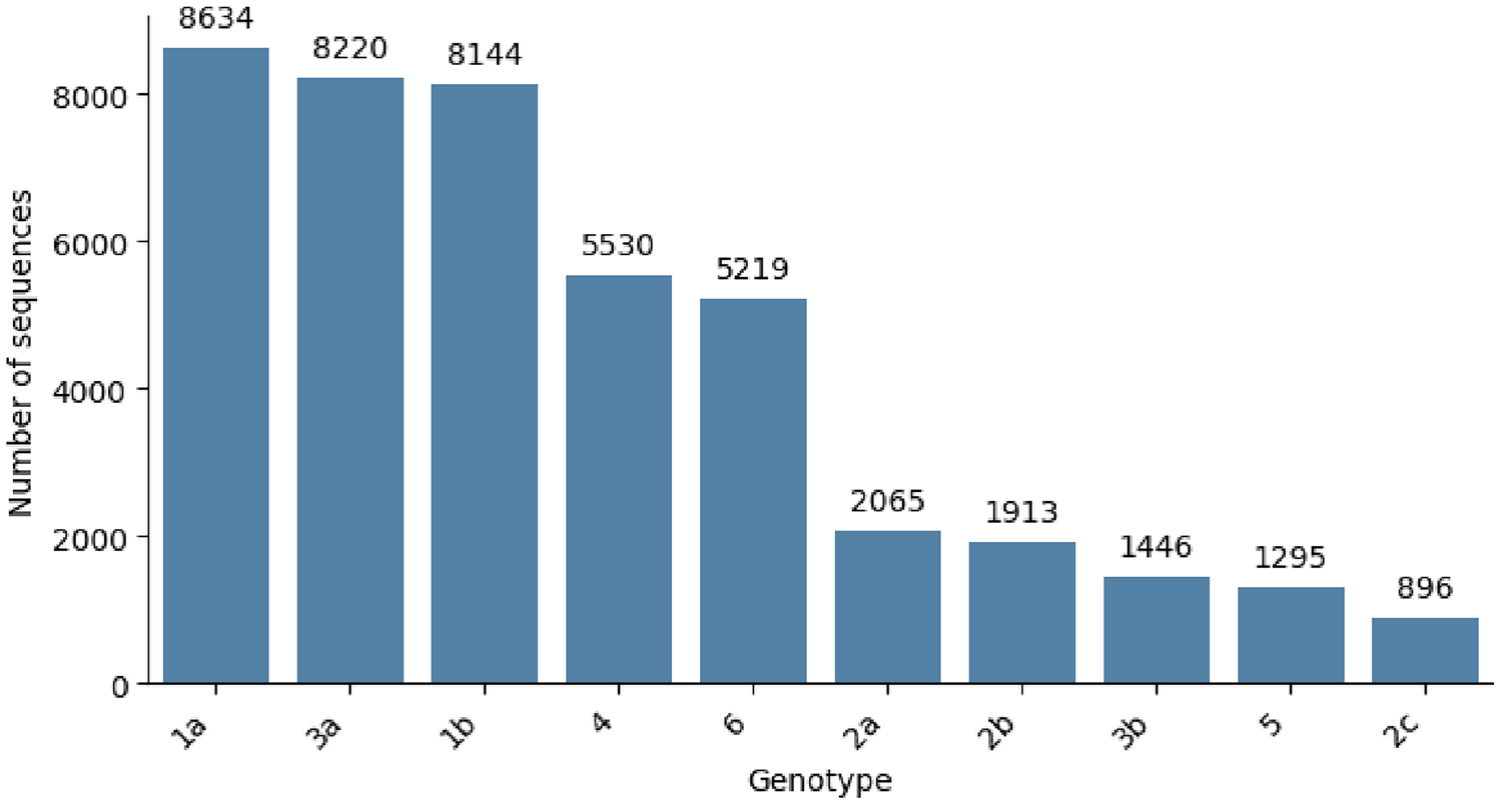
Distribution of each genotype in the dataset and the number of sequences in each genotype.

In this study, genomic sequences were collected as .fasta from The Los Alamos Hepatitis C Sequence Database^31,32^. The dataset encompassed a comprehensive compilation of both partial and complete sequences representing 10 distinct Hepatitis C Virus (HCV) genotypes, specifically 1a, 1b, 2a, 2b, 2c, 3a, 3b, 4, 5, and 6. Figure 2 illustrates the distribution of each genotype in the dataset.

### Genomic sequence preprocessing

Data preprocessing is a crucial machine learning step involving cleaning and transforming raw data into a format suitable for model training. Filtering out uncleaned samples during preprocessing is vital to model training as it removes noise and inconsistencies. In preprocessing, we analyzed the sequences to eliminate ambiguous nucleotides, retaining only the fundamental nucleotides (A, T, C, and G bases). The number of minimum and maximum sequence lengths of each HCV genotype are shown in Table 1.

**Table 1:** HCV genotypes distribution.

HCV genotype	Minimum and maximum lengths (nucleotides)
1a	54-9646
1b	60-9658
2a	60-9711
2b	81-9678
2c	164-9678
3a	60-9654
3b	104-9444
4	81-9597
5	192-9414
6	104-9641

As DNA sequences consist of categorical data, it is necessary to encode them into a numerical representation. The process of converting categorical nucleotide data into numerical form is known as encoding. In this study, Chaos Game Representation, Label encoding, and K-mer encoding techniques were employed to encode the DNA sequence, and their impact on classification performance is analyzed. The fundamental concept behind label encoding involves assigning a distinct integer to every category within a categorical variable. In our scenario, each nucleotide within the DNA sequence is allocated an integer value, with A, C, G, and T being assigned 1, 2, 3, and 4, respectively.

On the other hand, K-mer encoding is a technique used to transform a sequence of DNA into a fixed-length vector representation. It involves breaking the sequence into all possible subsequences of length k, where k is a predefined integer (K = 6 in our case, based on grid search). Each unique subsequence of length k is then represented as a feature in the vector, and its presence or absence in the sequence is used to encode the sequence. This technique is commonly used in bioinformatics for sequence classification and similarity analysis tasks. Chaos Game Representation will be discussed thoroughly in the next section.

### Mapping and feature extraction

#### Chaos game representation

Numerical encoding is a crucial aspect of computational DNA sequence analysis, where symbolic characters are associated with corresponding numerical values. Chaos Game Representation, introduced by Jeffrey^33^, is an iterative mapping technique that uniquely assigns each nucleotide in a DNA sequence to specific coordinates on a plane, allowing the representation of DNA sequences as images. Chaos Game Representation has been successfully applied in bioinformatics for over 30 years. This representation, resembling a 2D image of distributed dots in a 0-1 square matrix, offers unique properties of uniqueness and the ability to reverse the coordinates to their corresponding nucleotide or amino acid^34^. Employing graphical methods for investigating biological systems has proven its value, offering multiple insights across diverse realms of biological research. Numerous studies have applied graphical approaches to investigate critical topics, including DNA^29,30,35,36^, protein^37–42^, genome^43–46^, drug metabolism systems^47^, and protein-protein interactions^48^. In the CGR approach, each nucleotide in the sequence is mapped to a specific position in a 2D grid. The mapping is based on iterative processes that divide the grid into quadrants or other geometric shapes. For instance, considering a DNA sequence, each nucleotide (A, T, C, G) is associated with a specific geometric transformation. As the sequence is processed, a point in the grid is iteratively moved according to the rules associated with each nucleotide.

Frequency Chaos Game Representation (FCGR) is an extension of Chaos Game Representation that involves the counting of occurrences of specific nucleotide sequences or k-mers within the DNA sequence and their placement in the FCGR matrix^30^. This matrix provides a comprehensive view of the frequency distribution of these k-mers. A k-mer is a sequence of DNA or RNA nucleotides of length k. In genomics, k-mers are widely used for various applications such as sequence assembly, sequence alignment, and sequence analysis. The value of k determines the length of the k-mer, and different values of k can provide different insights into the structure and function of biological sequences. In the context of the frequency chaos game, k-mers are used to represent the frequency of occurrence of specific subsequences within a given sequence. By counting the occurrences of each k-mer in the sequence, we can create a frequency distribution that captures the local sequence composition. This information can then be visualized using the frequency chaos game representation, where each k-mer is assigned a specific position in a grid based on its frequency. This visualization provides a unique way to analyze and interpret the distribution of k-mers within a sequence, allowing for the identification of patterns and structures that may not be immediately apparent from the raw sequence data. In FCGR, the matrix elements correspond to the frequency of occurrence of each k-mer. By associating the frequency of k-mers with specific grid positions, FCGR provides a rich representation of the sequence occurrences of specific nucleotide sequences of varying lengths.

For example, to construct the first-order FCGR matrix (k = 1) for a given sequence *s*, we start with calculating the frequency of each nucleotide (C, G, A, and T) in the given sequence. This involves counting the occurrences of each nucleotide and then using these frequencies to construct a 2 × 2 matrix, where each cell represents the count of a specific nucleotide. The first-order FCGR matrix (k = 1) is given by the following equation:

1 $$\begin{aligned} FCGR_{1}(s) = \begin{pmatrix} f_{C} &{} f_{G}\\ f_{A} &{} f_{T} \end{pmatrix} \end{aligned}$$

**Figure 3:**
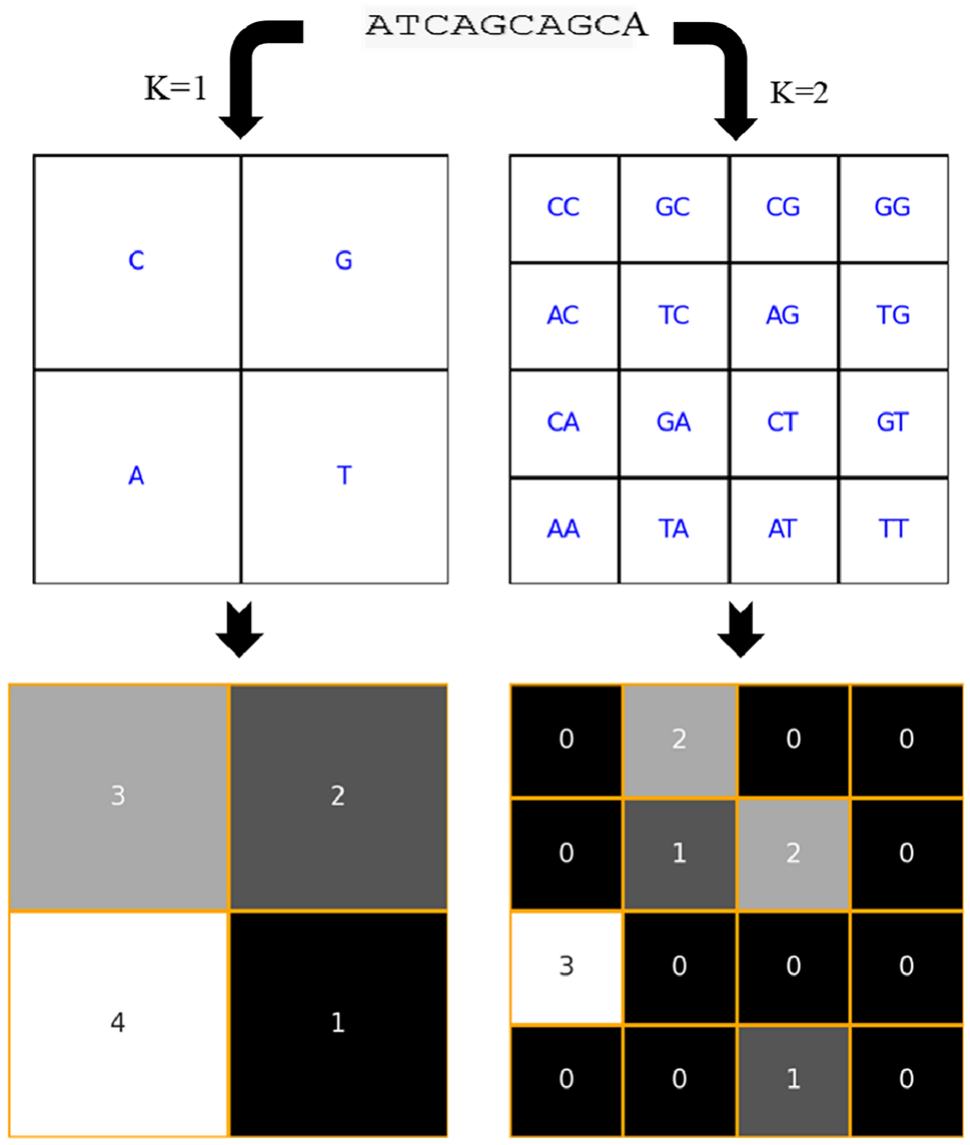
Frequency Chaos game representation matrices for varying kmers. On the left is the first-order FCGR (k = 1) and on the right is the second-order FCGR (k = 2).

The resulting image of the Frequency Chaos Game Representation (FCGR) is visualized using a grayscale color scheme. In this visualization, each cell in the 2D grid represents the count of a specific nucleotide or k-mers. The grayscale color scheme is employed to depict the frequency distribution of these k-mers within the sequence. The dimension of the resulting image is $2^{k} \times 2^{k}$, for example, the resulting image of the fifth-order FCGR matrix (k = 5) is $2^{5} \times 2^{5} = 32\times 32$ pixels. In the output image, Lighter shades of gray typically correspond to higher counts, while darker shades of gray indicate lower counts. This grayscale visualization effectively conveys the relative frequencies of the k-mers, enabling the identification of patterns and structures within the sequence data. The resulting image pixels are then normalized to be ready for the downstream task. Figure 3 illustrates the FCGR algorithm of an input sequence.

**Figure 4:**
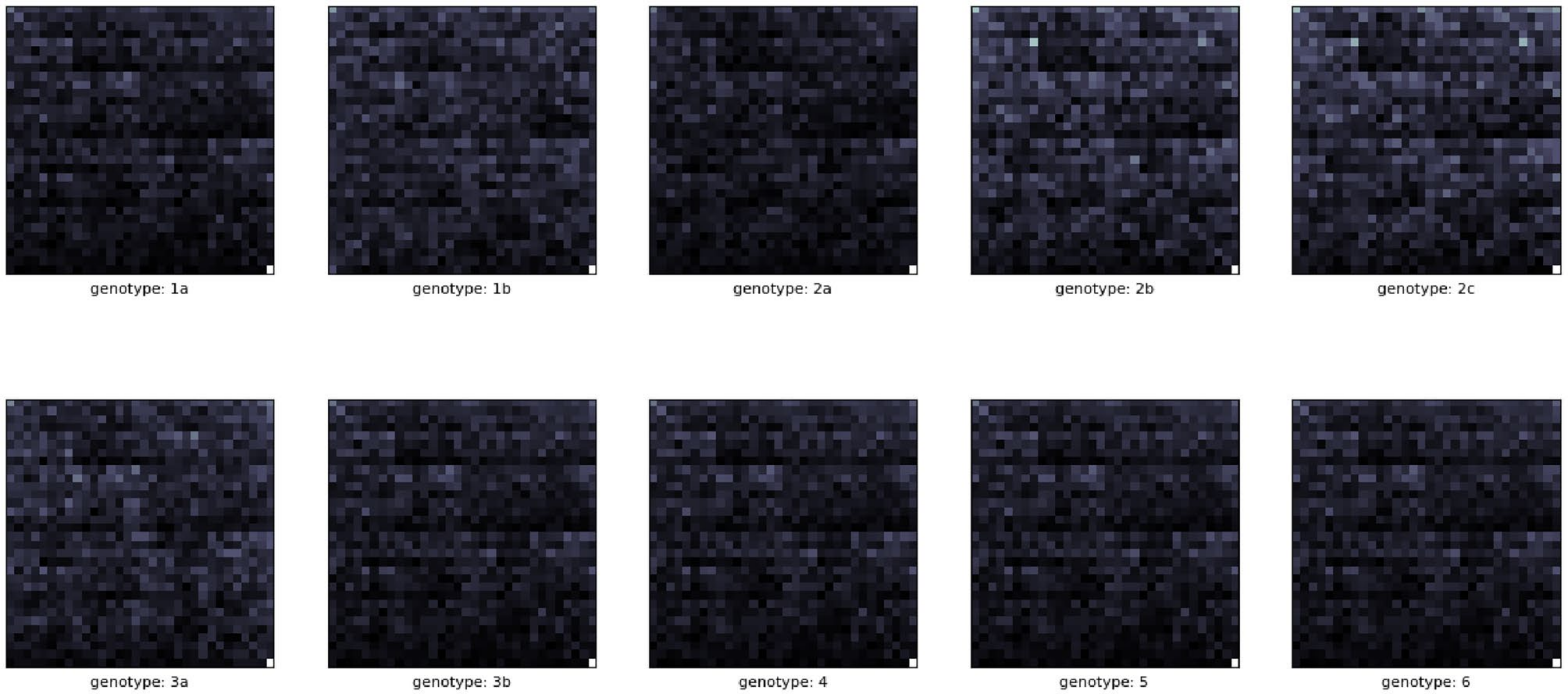
Fifth-order Frequency Chaos Game Representation from different HCV genotypes.

Figure 4 displays the Fifth-order Frequency chaos game representations of the coding sequences from different HCV genotypes.It is obvious that these different genotypes tend to have different patterns.

#### Autoencoder as self-supervised

Self-supervised learning (SSL) is a training approach in Deep Learning (DL) that enables models to classify images without predefined labels during training. SSL is significant because it trains DL models to extract optimal features from images without relying on explicit labels^49^. Unlike supervised learning, where models are constrained by predefined labels, SSL allows models to understand sensory signals, like object shapes, directly from input images. This approach frees models from discrete representations, enabling richer feature extraction and more effective grouping of images from the same class based on patterns and features learned from the data. Recent research highlights SSL’s effectiveness in enhancing feature extraction and pattern recognition capabilities in DL models.

Self-supervised learning methods rely on an Encoder, also known as a feature extractor, which plays a pivotal role in extracting essential features from images. The Encoder filters out noise and transforms these features into a vector representation that can be projected into an n-dimensional or latent space. In this space, images from the same class are clustered together, facilitating effective differentiation between classes. Encoders are trained to enhance their representation of images by encoding richer features into vectors that enable distinct clustering. These encoded vectors can be utilized for various computer vision tasks, such as image classification. An example of self-supervised learning is the Autoencoder, which functions as both an encoder and a decoder. The encoder compresses input data into a latent space, and the decoder reconstructs the input from this compressed representation. During training, the goal is to minimize reconstruction error without relying on external labels, forcing the model to capture meaningful features during compression and accurately reconstruct the original input during decoding. This self-supervised approach allows the model to learn representations without explicit supervision, making it versatile for unsupervised feature learning.

The structure of the autoencoder, comprises three standard types of layers found in any neural network: input, hidden, and output layers. However, in this case, the values in the input and output nodes are kept the same.

Although deep convolutional neural networks have demonstrated remarkable performance in image classification, they are susceptible to noise and redundant information in high-dimensional raw images, which can result in unreliable predictions. In this study, we introduce a convolutional autoencoder model to mitigate the vulnerability of deep learning models. Specifically, the model is intended to compress and remove redundant information and noise from the high-dimensional input images obtained from Frequency the Chaos Game Representation. The compressed output is then fed into a classification model. Our results show that this strategy works well, producing a notable improvement in performance when compared to deep learning models trained using the raw images from FCGR.

**Figure 5:**
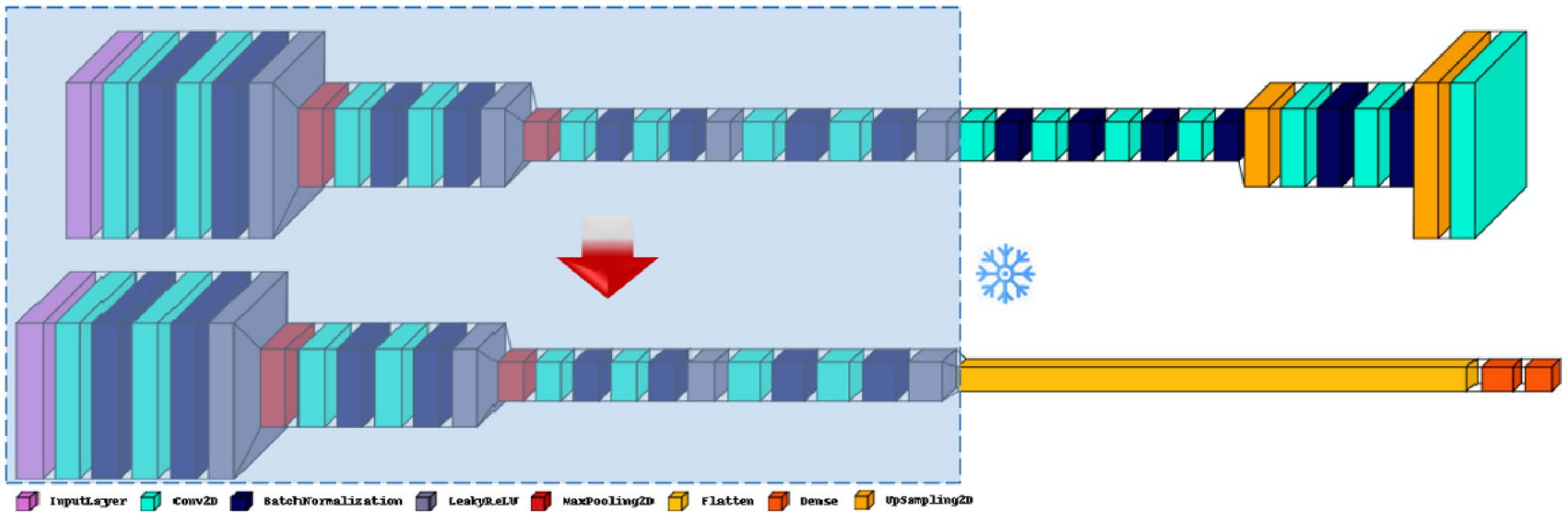
The above architecture shows the architeccture of the autoencoder and the bottom one shows the architecture of the CNN. The encoder part is frozen and used as the base model.

Figure 5 shows the proposed model, encompassing the autoencoder with convolutional layers for both the encoder and decoder. The encoder consists of four sets of convolutional layers with increasing filter sizes (32, 64, 128, 256) and max pooling layers to downsample the feature maps. Each convolutional layer is followed by batch normalization and LeakyReLU activation. The decoder consists of three sets of convolutional layers with decreasing filter sizes (128, 64, 32) and upsampling layers to upsample the feature maps. The final layer uses a convolutional layer with a sigmoid activation function to produce the decoded output (all the hyperparameter is set based on grid-search tuning).

### Cross validation

In this work, a tenfold cross-validation approach was employed to evaluate the performance of the classification model for the 10 HCV genotypes. Stratified cross-validation ensured an even distribution of genotypes in each fold. Randomly generated indices prevented bias in training and validation set selection, providing robust and representative results. This approach allowed for comprehensive model performance evaluation and guided the selection of the best-performing model.

### Classification

In this study, we compared our proposed model with five different deep learning models utilizing different encoding methods. Our classification algorithms for the first three models, primarily rely on Convolutional Neural Networks (CNN). *CNN*, a widely used deep-learning method, has demonstrated state-of-the-art performance in various classification tasks. CNNs are widely used in medical research, speech recognition, computer vision, and NLP and have consistently delivered impressive outcomes. CNNs have particularly excelled in tasks such as image classification, localization, and segmentation in medical image processing^50–53^, and multiple studies have employed CNN for viral classification^54–57^. Unlike traditional machine learning models where feature selection is manual, CNN automatically extracts features from the input data. 2D CNN is commonly used for image data. The CNN architecture consists of convolutional layers followed by max-pooling layers, which reduce the dimensions of the extracted features. Different types of hyperparameters play a crucial role in the performance of the model, like the number of layers, the number of neurons in each layer, and the size of the kernel in the convolutional layer^58^.

For the other two models, we extended our comparison to include two additional recurrent neural network (RNN) architectures: the Gated Recurrent Unit (GRU) and Long Short-Term Memory (LSTM) models. In the proposed approach, CNN is employed for the classification of the deep features extracted from the autoencoder. Figure 5 shows the complete architecture of the proposed model used in this work. We use the encoder part of the autoencoder as our base model. First, we freeze the encoder layers up to the seventh Convolutional layer, as illustrated in the figure. Finally, the flattened layer converts the 3D feature maps into a 1D array with 16,384 elements. This is followed by two Dense layers, the first with 256 neurons and the second is the output layer with ten neurons. The softmax function is used to compute the probability of each class.

## Results and discussion

### Implementation details

The proposed models were implemented using NVIDIA T4 with a RAM size of 16 GB. The dataset consists of 43,362 sequence data of both complete and partial sequences. The dataset is divided into training, validation, and testing ratios of 70%, 10%, and 20%, respectively. The training, validation, and testing sets consist of 31,220, 3469, and 8673 samples, respectively, with a maximum sequence length of 9711. We compared our proposed model with five deep learning models of different encoding methods as follows: CNN on raw images where DNA sequences were encoded into images using FCGR, CNN with label encoding, CNN, GRU and LSTM with K-mer encoding. The proposed model is implemented as CNN with autoencoder-based feature extraction (auto-CNN) where DNA sequences were encoded into images using FCGR. The comparison is done by varying the values of different hyperparameters, such as the number of layers and filters. Cross-validation is used to select the best combination of hyperparameters.

**Table 2:** Performance comparison of the CNN with autoencder as feature extraction and different CNN models without feature extraction.

Model		Genotype										
		1a	1b	2a	2b	2c	3a	3b	4	5	6	
CNN	Accuracy	0.99	0.99	0.98	0.98	0.97	1	1	0.94	1	0.92	0.977
	Sensitivity	0.99	0.98	0.98	0.96	0.93	0.99	0.96	0.94	0.98	0.94	0.97
	Specificity	0.99	0.99	0.99	0.99	0.99	0.99	0.99	0.99	0.99	0.99	**0.99**
	Precision	0.98	0.98	0.93	0.94	0.92	0.99	0.95	0.98	0.96	0.98	0.96
	F1-score	0.98	0.98	0.95	0.95	0.93	0.99	0.96	0.96	0.97	0.96	0.96
Auto-CNN	Accuracy	0.99	1	1	1	1	1	1	0.99	0.99	1	**0.997**
	Sensitivity	1	1	0.99	0.99	0.98	1	0.99	1	0.99	0.99	**0.99**
	Specificity	0.99	0.99	0.99	0.99	0.99	0.99	0.99	0.99	0.99	0.99	**0.99**
	Precision	1	1	0.99	1	0.99	1	0.99	0.99	1	0.99	**0.99**
	F1-score	1	1	0.99	1	0.98	1	0.99	0.99	0.99	0.99	**0.99**
Kmer-CNN	Accuracy	0.97	1	0.97	1	1	1	0.98	0.92	1	0.85	0.969
	Sensitivity	1	0.99	0.97	0.98	0.93	0.99	0.98	0.99	0.99	0.97	0.98
	Specificity	0.99	0.99	0.99	0.99	0.99	0.99	0.99	0.99	0.99	0.99	**0.99**
	Precision	0.98	0.99	0.99	0.93	0.97	1	0.98	0.99	1	1	0.98
	F1-score	0.99	0.99	0.98	0.96	0.95	0.99	0.98	0.99	0.99	0.98	0.98
Labelencoding-CNN	Accuracy	0.99	0.94	0.99	1	0.95	1	0.99	0.93	0.99	0.97	0.975
	Sensitivity	0.99	0.98	0.97	0.93	0.91	0.97	0.96	0.95	0.94	0.96	0.96
	Specificity	0.99	0.99	0.99	0.99	0.99	0.99	0.99	0.99	0.99	0.99	**0.99**
	Precision	0.98	0.98	0.96	0.96	0.98	0.98	0.9	0.94	0.99	0.96	0.96
	F1-score	0.99	0.98	0.96	0.94	0.94	0.97	0.93	0.95	0.96	0.96	0.96
Kmer-LSTM	Accuracy	0.98	0.97	0.96	0.98	0.99	0.97	0.94	0.98	0.99	0.98	0.974
	Sensitivity	0.99	0.99	0.98	0.98	0.96	0.99	0.98	0.98	0.99	0.97	0.98
	Specificity	0.99	0.99	0.99	0.99	0.99	0.99	0.99	0.99	1	0.99	**0.99**
	Precision	0.99	0.97	0.97	0.98	0.97	0.99	0.98	0.99	1	0.99	0.98
	F1-score	0.99	0.98	0.98	0.98	0.96	0.99	0.98	0.98	0.99	0.98	0.98
Kmer-GRU	Accuracy	0.98	0.97	0.97	0.97	0.98	0.97	0.95	0.97	0.98	0.98	0.975
	Sensitivity	0.99	0.99	0.98	0.98	0.96	0.99	0.98	0.98	0.99	0.97	0.98
	Specificity	0.99	0.99	0.99	0.99	0.99	0.99	0.99	0.99	1	0.99	**0.99**
	Precision	0.99	0.97	0.97	0.98	0.97	0.99	0.98	0.99	1	0.99	0.98
	F1-score	0.99	0.98	0.98	0.98	0.96	0.99	0.98	0.98	0.99	0.99	0.98

The best results are boldfaced.

The best hyperparameter combination of the CNN is 32 and 64 filters, each of size 3 × 3 in the convolutional layers and 256 in the dense layers of the model. Whereas for the label encoding and K-mer encoding CNN, the best hyperparameter combinations are 32 and 64 filters, each of size 3 in the convolutional layers and 256 in the Dense layers.

For the RNN models, The GRU model is configured with a GRU layer of 128 units followed by a dense layer with 64 units. Similarly, the LSTM model includes an LSTM layer with 50 units configured for sequence processing, followed by a dense layer with 64 units. The input to RNN models is encoded using k-mer encoding (k = 6 in our case, set by grid search).

For the proposed model, auto-CNN was implemented by considering the encoder part of the autoencoder as our base model, which consists of four convolutional blocks. Each block contains two convolutional layers with batch normalization and ReLU activation, followed by a max-pooling layer. The first convolutional block has 32 filters, the second has 64 filters, the third has 128 filters, and the fourth has 256 filters. Each convolutional layer uses a 3 × 3 kernel size. Additionally, the LeakyReLU activation function is applied after the second convolutional layer in each block. We add Dense layer of 256. All the layers up to the seventh convolutional layer were frozen, and the whole model was trained. All the models were trained for 10 epochs on the whole training set except for the auto-CNN, While the autoencoder part used for feature extraction was trained first for 40 epochs. Auto-encoders have proven efficiency in addressing imbalanced datasets thanks to their ability to learn compact representations of input data. By leveraging the reconstruction process, auto-encoders can learn underlying patterns and features within the data, making them adept at handling skewed class distributions. This approach allows for improving model performance and mitigating bias towards dominant classes.

### Efficiency and performance comparison

Table 2 shows the performance comparison of the implemented models. The proposed model outperforms the simple versions of the CNN with different encoding methods. The performance of the proposed approach was evaluated using the following four metrics: sensitivity (SEN), specificity (SPEC), precision (PREC), accuracy (ACC), and F1-score (F1), Matthew’s Correlation Coefficient (MCC). Based on the analysis, auto-CNN model, which uses FCGR encoding, achieves the highest performance in all metrics among the other three models. Notably, it excels in addressing data scarcity in underrepresented genotypes such as genotype 2c and the notoriously error-prone like genotype 6. The best results are boldfaced. Although kmer-CNN and labelencoding-CNN utilize the same model architecture, the labelencoding-CNN model tends to exhibit higher performance. This observation suggests that the encoding method plays a significant role in determining the model’s performance. The differences in performance between these models indicate that the choice of encoding method can affect the model’s ability to effectively learn and extract meaningful patterns from DNA sequences. This highlights the importance of choosing an appropriate encoding method adapted to the characteristics of the input data, as this can directly affect the model’s performance.

**Figure 6:**
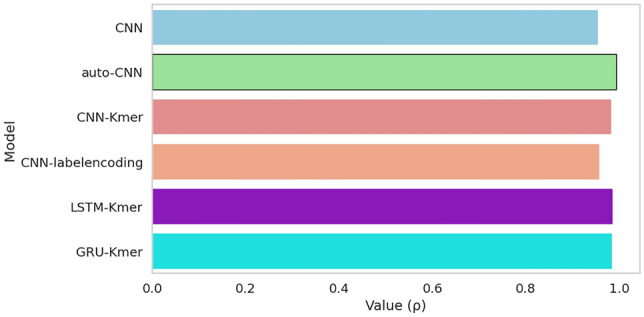
Correlation levels of different models including, CNN, auto-CNN, Kmer-CNN, labelencoding-CNN, Kmer-LSTM and Kmer-GRU. Auto-CNN which leverages autoencoder as feature extractor outperforms all the models.

The correlation coefficient results shown in Fig. 6 illustrates that the proposed model achieved high correlation level of 0.99 outperforming other models having correlation levels in range of 0.95–0.98. Overall, this is due both the encoding method and the effectiveness of the deep features extracted by the autoencoder.

**Table 3:** Comparison of the model capacity and execution time.

Model	Number of parameters (million)	Size (MB)	Training time (min)	Inference time (s)
CNN	0.61	2.33	0.85	1.06
auto-CNN	5.37	20.50	5.5	1.28
kmer-CNN	9.38	35.79	2.13	2.17
labelencoding-CNN	9.09	34.66	2.45	3.11
Kmer-LSTM	3.21	12.26	25.55	20.73
Kmer-GRU	3.25	12.43	25.66	21.21

**Table 4:** Comparison of performance of classical machine learning models.

Model	Accuracy
SVM	0.62
KNN	0.71
LR	0.75
RF	0.83

Table 3 presents a comparison of the six models based on various metrics. The standard CNN stands out for having significantly fewer parameters (0.61 million) compared to the auto-CNN (5.37 million), kmer-CNN (5.74 million), labelencoding-CNN (9 million), Kmer-LSTM (3.21 million) and Kmer-GRU (3.25 million). This results in a smaller size of 2.33 MB for the standard CNN, compared to 20.50 MB for the auto-CNN, 34.66 MB for labelencoding-CNN, 12.26 MB for Kmer-LSTM, 12.43 MB for Kmer-GRU and 35.79 MB for Kmer-CNN, which is considered the largest among them. The standard CNN is notably more lightweight and requires less memory storage. However, although the sizes of auto-CNN, Kmer-CNN and labelencoding-CNN are larger than the standard CNN, they remain small in terms of a few megabytes; in addition, the larger number of parameters and size allow them to capture more complex patterns and features in the data, leading to better performance.

In terms of training duration, the CNN models using label encoding and k-mer encoding require 2–3 min, whereas kmer-LSTM and kmer-GRU models have the longest training time, approximately 25 min. The standard CNN completes training in less than 1 min, representing the shortest duration, this is a result of the smallest its smallest size. However, the auto-CNN model takes about 25 min for training, split into 20 min for training the autoencoder and 5 min for training the classifier. These diverse training times underscore the computational demands and complexities associated with different model architectures and training processes. However, when it comes to inference time, the models perform differently, with the standard CNN and auto-CNN taking around 1.06–1.28 s, the kmer-CNN taking 2.17 s and labelencoding-CNN taking 3.11 s, respectively. You can observe that the inference time of auto-CNN is roughly the same (less) as the light-weighted CNN. While RNN models, both LSTM and GRU takes the longest inference time among all the models.

Our analysis reveals that The variation in the number of parameters and training time between LSTM/GRU models and CNNs comes from fundamental differences in their architectures and computational demands. CNNs typically exhibit higher parameter counts due to the expansive connectivity patterns of convolutional layers, which involve numerous filters. In contrast, LSTM and GRU models emphasize recurrent connections and hidden states. The computational complexity of LSTM and GRU models is heightened by their sequential processing requirements over time steps, contrasting with the parallel nature of CNN operations. Furthermore, CNNs benefit from efficient parallelization during training, particularly with GPU utilization, which accelerates training speed. Ultimately, these differences reflect the distinct characteristics and computational needs inherent to each model type, with CNNs excelling in spatial data tasks while LSTM/GRU models are suited for sequential data but may demand more resources and time for training. In the comparison of the classical machine learning models, we utilized the label-encoding approach to encode the input data. The models evaluated includes, k-Nearest Neighbors (KNN), Random Forest (RF), LogisticRegression (LR) and Support Vector Machine (SVM). Table 4 presents a performance comparison of the models, with accuracies of 62%, 71%, 75% and 83% achieved by SVM, KNN, LR, and RF respectively. Classical machine learning models have shown inefficiency in achieving higher accuracies compared to more sophisticated models. This indicates the limitations in capturing the underlying relationships within the dataset.

**Figure 7:**
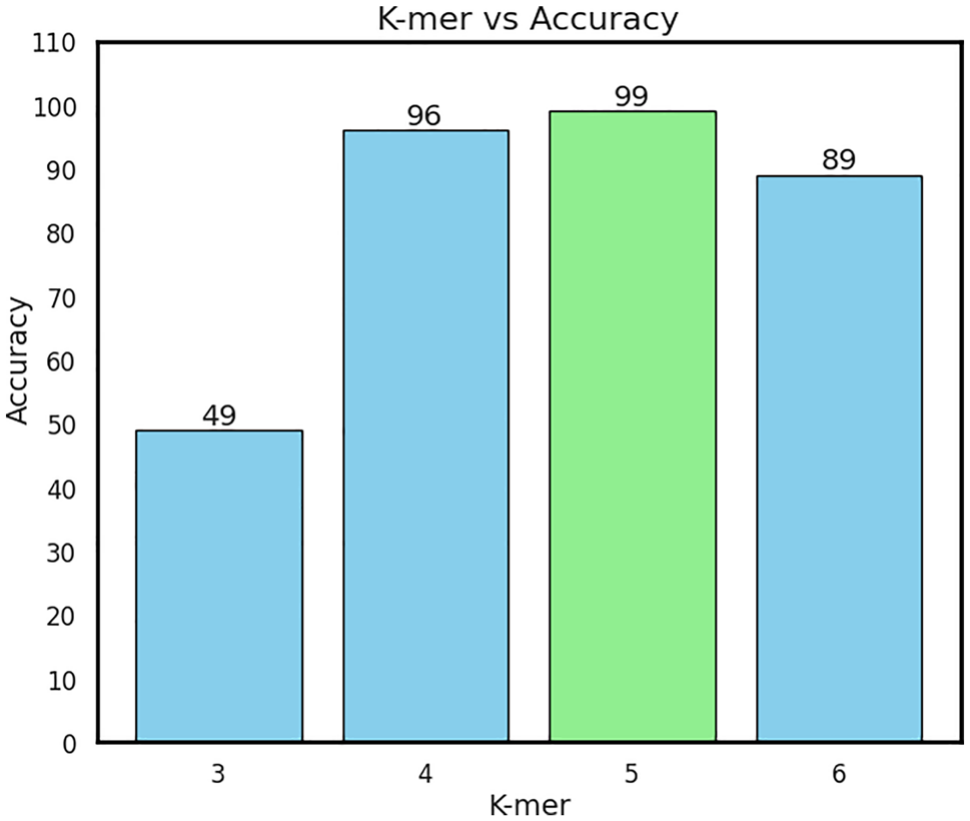
Comparison of different K-mer values in CGR and the effect of the model performance.

### Effect of K-mer size

From another perspective, the performance of our model on HCV genomic sequences is notably impacted by the choice of k-mer value used in the encoding process of CGR. From Fig. 7, it is clear that the performance of the model appears to be significantly influenced by the choice of k-mer size in the encoding method. Across different k-mer values tested (k = 3, k = 4, k = 5, k = 6), we observed varying levels of accuracy. Notably, a k-mer size of 5 resulted in the highest accuracy, achieving 99%, followed closely by k = 4 with 96% accuracy. In contrast, using k = 3 or k = 6 led to notably lower accuracy scores of 49% and 89%, respectively. These findings suggest that the selection of k-mer size plays a critical role in determining model performance, with optimal accuracy achieved at intermediate k-mer lengths. This emphasize the importance of selecting an optimal k-mer value to enhance model effectiveness in analyzing HCV genomic sequences, with appropriate adjustments crucial for improving predictive accuracy and model robustness in computational biology applications.

### Effect of sequence length

**Figure 8:**
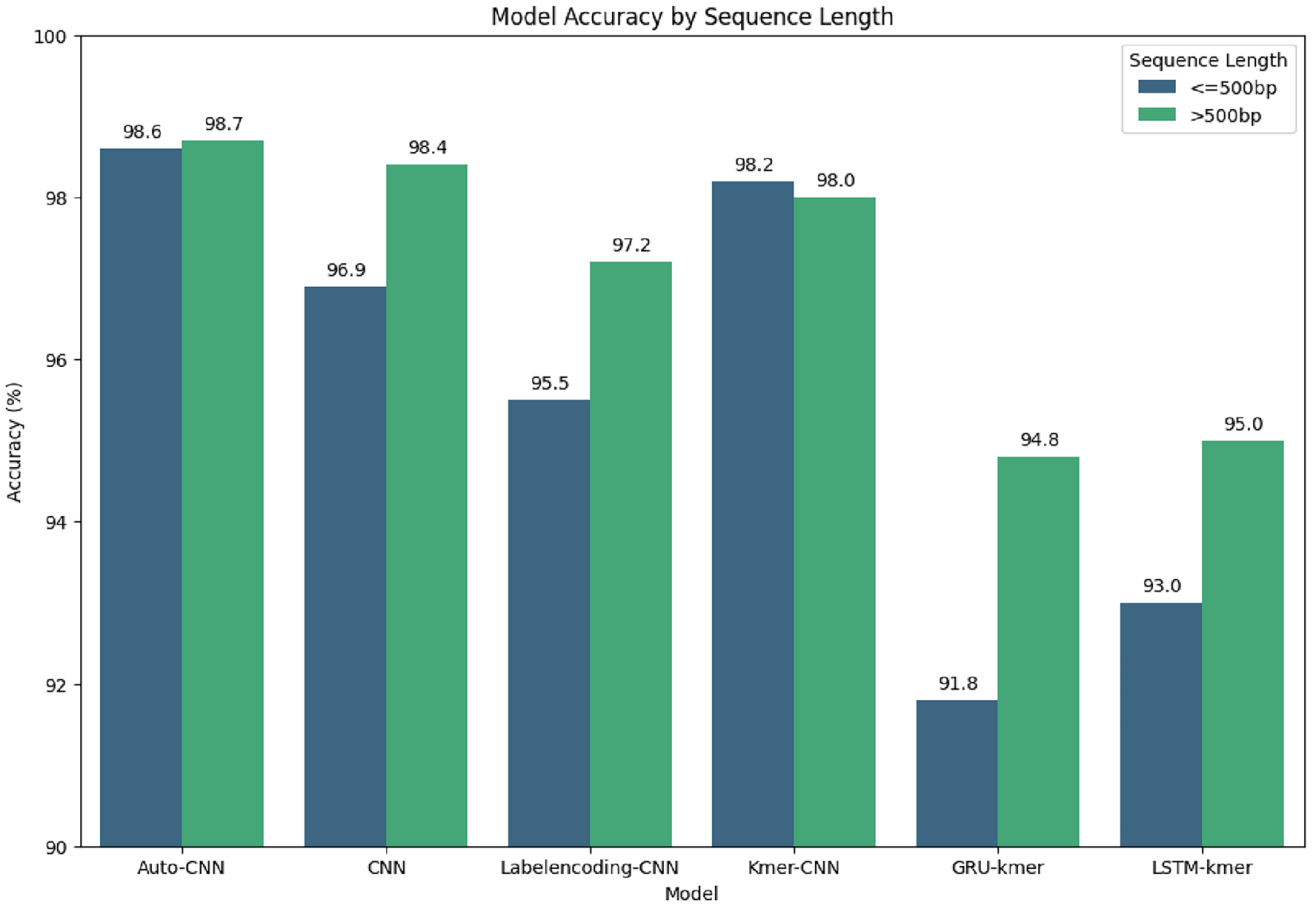
Analysis on the effect of input sequence length on the accuracy score.

Figure 8 shows the effect of the sequence length reveals a clear trend, longer HCV sequences lead to improved accuracy across almost all models, which is advantageous for model performance. This pattern is consistent across various architectures, including Auto-CNN, standard CNN, Labelencoding-CNN CNN, Kmer-CNN, GRU-kmer, and LSTM-kmer models. The trend indicates that longer sequences provide more comprehensive information, allowing the models to make more accurate predictions. This improvement in accuracy with longer sequences highlights the models’ ability to better capture the complexities in the data, ultimately leading to more reliable and effective genotyping. Overall, the positive impact of longer HCV sequences on model accuracy highlights the importance of utilizing extended sequence lengths in viral genotyping tasks.

### Related methods

The NCBI viral genotyping tool^59^ is a web-based application used to determine the genotype of viral sequences by sliding a window along the query sequence and using BLAST to compare each segment to reference sequences. It assigns the genotype with the highest similarity score to each segment, then combining results to determine the overall genotype. However, this tool is limited by its reliance on sequence alignment, which is less effective for sequences without significant homology and is time-consuming for large-scale tasks^29^. However, the proposed method employs Chaos Game Representation to provide a scale-independent representation of DNA sequences through statistical nucleotide distribution. In addition, it eliminates the need for database sequence searches. Table 5 shows the output accuracy of the NCBI genotyping tool. It is clear that it fails to perfectly align the input sequence to the right substype.

**Table 5:** Accuracy results of NCBI genotyping tool on the dataset.

Subtype	Accuracy
1a	0.84
1b	0.77
2a	0.90
2b	0.87
2c	0.79
3a	0.76
3b	0.94
4	0.91
5	0.94
6	0.97

## Conclusion

This study unveils the viable application of self-supervised learning as a feature extractor in the field of viral genomics. We introduce a powerful deep learning approach and a comprehensive baseline for HCV genotyping by employing convolutional auto-encoders on genomic data. We have shown its exceptional performance compared to traditional classification methods, effectively addressing the challenge of data scarcity in genomic data. Our systematic analysis demonstrates its strong performance and competitive inference latency. The model’s accuracy exceeds 99%, demonstrating the efficacy of the autoencoder in extracting significant features from the fifth-order FCGR images. Furthermore, we have demonstrated that the proposed model holds significant weight from one perspective and offers rapid and accurate inference compared to traditional models from another perspective. Furthermore, our investigation into input encoding revealed that k-mer encoding yields notably better performance compared to label encoding on the same model, leading us to the conclusion that the encoding method affects the performance of the model. We also examined the effect of different K-mer lengths in the CGR across varying k-mer values and observed significant differences in accuracy. We highlighted that the most effective k-mer value depends on the specific characteristics and complexities of the genomic data being analyzed. This emphasizes the need for a tailored approach in selecting the appropriate k-mer size to achieve optimal model performance and accuracy in computational biology studies.

### Supplementary Information

Supplementary Information.

## Data Availability

The genome sequences used in this study are available in the Los-Alamos repository, https://hcv.lanl.gov/content/sequence/HCV/ToolsOutline.html, and the accession numbers of these sequences are available as supplementary material.
